# Bacteriophage-Gated Optical Sensor for Bacteria Detection

**DOI:** 10.1021/acs.analchem.5c00780

**Published:** 2025-05-23

**Authors:** Busra Canan Aslan, Esra Ekiz, Emine Kubra Tayyarcan, Ismail Hakki Boyaci, Aysen Gumustas, Ender Yildirim, Ugur Tamer, Ebru Evren, Elcin Ezgi Ahi, Ramón Martínez-Máñez, Mehmet Gokhan Caglayan

**Affiliations:** † Faculty of Pharmacy, Department of Analytical Chemistry, Ankara University, Ankara 06560, Türkiye; ‡ Graduate School of Health Sciences, Ankara University, Ankara 06560, Türkiye; § Department of Food Engineering, Hacettepe University, Ankara 06800, Türkiye; ∥ Faculty of Pharmacy, Department of Pharmaceutical Microbiology, Ankara University, Ankara 06560, Türkiye; ⊥ ODTU MEMS Center, Ankara 06530, Türkiye; # Department of Mechanical Engineering, Middle East Technical University, Ankara 06800, Türkiye; ∇ Faculty of Pharmacy, Department of Analytical Chemistry, Gazi University, Ankara 06500, Türkiye; ○ School of Medicine, Department of Medical Microbiology, Ankara University, Ankara 06560, Türkiye; ◆ Department of Chemistry, Gebze Technical University, Kocaeli 41400, Türkiye; ¶ Instituto Interuniversitario de Investigación de Reconocimiento Molecular y Desarrollo Tecnológico (IDM), Universitat Politècnica de València, Universitat de València, Camino de Vera s/n, València 46022, Spain

## Abstract

Bacterial infections significantly impact public health, and the growing threat of antimicrobial resistance underscores the urgent need for rapid and accessible diagnostic tools. This study presents a novel bacteriophage-gated sensor for the selective detection of . The sensor utilizes sulforhodamine B as a fluorescent reporter dye encapsulated in mesoporous silica particles, with bacteriophages acting as gatekeepers. Upon interaction with , the bacteriophages detach from the silica surface, releasing the dye and producing a measurable fluorescence signal. The sensor demonstrated high sensitivity for detection, with a detection limit of 10^1^ CFU/mL achieved within 5 min. Integrated into a Point-of-Care Testing device, the system aligns with ASSURED criteria, offering affordability, sensitivity, specificity, and ease of use. The fluorescence response is detectable even by the naked eye using a simple LED lamp, enabling nonprofessional operation. Validation with real water samples confirmed the sensor’s accuracy, with results consistent with standard culture-based methods. This bacteriophage-gated sensor provides a low-cost, rapid, and reliable tool for bacterial detection with potential applications in environmental monitoring and clinical diagnostics.

## Graphical abstract

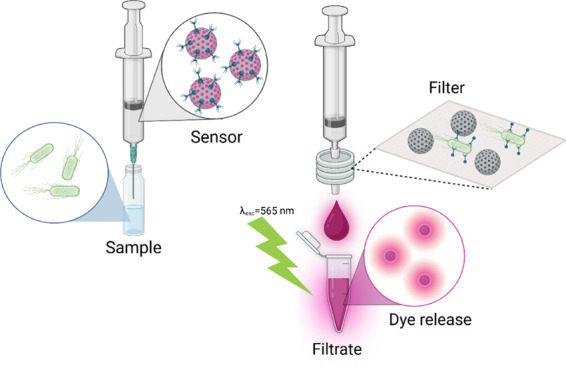

## Introduction

Bacterial infections significantly affect public health, causing disease at various body sites through either the bacteria themselves or the body’s response to them. Transmission to humans occurs via air, water, food, or living carriers.^1^ Moreover, antimicrobial resistance (AMR) is considered a major threat to global health and development. In 2019, bacterial AMR was estimated to have directly caused 1.27 million deaths worldwide and played a role in 4.95 million deaths overall.^2^ Key priorities for tackling AMR in human health involve preventing infections to reduce unnecessary antimicrobial use, providing universal access to reliable diagnoses and suitable treatments, and promoting strategic information and innovation. This includes AMR and antimicrobial usage surveillance, along with advancing research and development for new vaccines, diagnostics, and medicines.^3^ There are several detection methods for bacterial infections, such as culture tests, nucleic acid probes and polymerase chain reaction (PCR) procedures, serological tests, and antigen detection. Moreover, a number of research groups have been working on the development of rapid, on-site, and accessible testing methods like Lateral Flow Assays,^4^ paper-based devices,^5,6^ plasmonic sensors,^7^ smartphone-based sensors,^8^ etc. Among these on-site testing systems, Lateral Flow Immunoassay strips have been commercialized and used in daily life, since they do not require any instrumentation or training for use. Although these test strips achieve high sensitivity and specificity values at high concentrations, their performance may be reduced at low concentrations. For instance, in a study by Stapleton et al., sensitivity values of Lateral Flow Immunoassay strips were found to be 99%, 96%, and 86% for urine samples containing Gram-negative bacteria at concentrations of ≥10^5^ CFU/mL, ≥10^4^ CFU/mL, and ≥10^3^ CFU/mL, respectively, with specificity measured at 94%.^24^ In addition, Lateral Flow test strips often experience significant analyte loss during flow. Sensitivity issues, as seen in the example above, may arise due to analytes either adhering to the paper material without reaching the test line or passing through the test line without binding. Additionally, antibodies are usually used as analyte-selective agents in Lateral Flow test strips. While antibodies are useful in these systems due to their high analyte specificity, they have disadvantages, including sensitivity to pH, temperature, and redox reactions, lengthy and costly production processes, and activity variations across different production batches.^9^ On the other hand, bacteriophages, commonly known as phages, are good alternatives for bacterial tests due to their ability to be isolated from natural materials, ease of reproduction, low cost, and high stability. Bacteriophages are viruses that specifically infect bacteria and are the most prevalent microorganisms in nature. They play a crucial role in maintaining the balance of microbial ecosystems.^10^

Bacteriophages have been utilized as specific receptors in various analytical sensing techniques^11–13^ however, their application in gated sensors has yet to be explored. Gated sensing systems typically consist of a porous inorganic support with pores filled with dyes and a gatekeeper immobilized on the support surface to retain the dyes. Upon interaction with the target, the gatekeeper detaches from the support, resulting in the release of dyes from the pores. This method is known for its high sensitivity, as a single target can trigger the release of numerous dye molecules.^14,15^ In this study, we investigate the use of bacteriophages in gated sensors for the first time (Figure 1). Specifically, the bacteriophages infecting K12 were employed as gate materials to target K12 bacteria, and the analytical setup was adapted for a Point-of-Care Testing (PoCT) system using a simple injector and a reusable filter cartridge. The PoCT system was tested on lake water samples, and the results were validated against routine bacterial culture testing.

**Figure 1:**
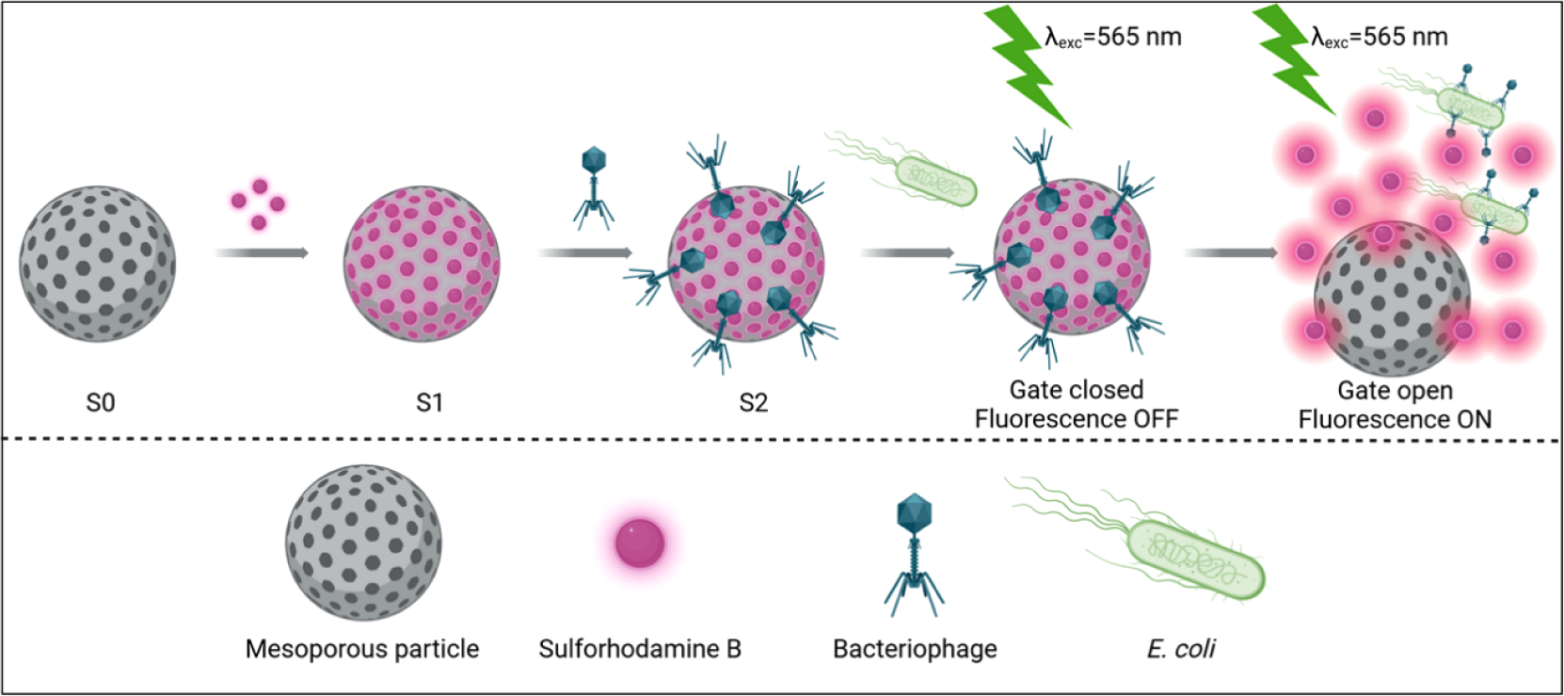
Schematic representation of the synthesis and response mechanism of bacteriophage-gated porous silica particles.

## Experimental Section

### Synthesis of Mesoporous Silica Particles

Mesoporous silica nanoparticles (∼100 nm in size) and mesoporous silica microparticles (∼1.7 μm in size) were synthesized and evaluated for their performance. The synthesis of MCM-41 mesoporous silica nanoparticles followed the procedure described by Tao et al.^16^ For mesoporous silica microparticles, this method was modified as follows:

Briefly, 11.0 mmol of CTAB was dissolved in 960 mL of distilled water. Subsequently, 14 mL of 2.0 M NaOH solution was added, and the mixture was stirred at 80 °C for 30 min. Then, 101.2 mmol of TEOS was gradually added to the reaction solution, which was stirred for an additional 2 h at 80 °C. The resulting mixture was filtered while still hot, and the solid product was thoroughly washed with deionized water and ethanol. The precipitate was dried overnight at 80 °C to obtain the “as-synthesized” mesoporous silica microparticles.

To prepare the mesoporous silica microparticles for experimental use, the “as-synthesized” material underwent partial calcination at 200 °C for 5 h. These partially calcined microparticles (**S0**) were then used in subsequent experiments.

### Bacterial Strains, Bacteriophage, and Growth Media

 K12 ( K12), ATCC 19948 (), enterica subsp. enterica serovar Enteritidis ATCC BAA-1045 (), and () used in the present study were obtained from the culture collection of the Boyaci Research Group, Department of Food Engineering, Hacettepe University, Türkiye. The bacteriophage K12.4a was isolated and characterized within the scope of a previous study.^17^ Tryptic Soy Broth (TSB), Tryptic Soy Agar (1.5%), and Tryptic Soy Soft Agar (0.6%) were used for the growth of bacteria, propagation of bacteriophages, and determination of the bacteriophage titer, and were purchased from Merck (Darmstadt, Germany). Eosin Methylene Blue (EMB) agar (Merck) was used for the enumeration of . Bacteria and bacteriophage stocks were prepared with 30–40% (v/v) glycerol (Merck) and stored at −18 °C during the study.

The bacteriophage K12.4a was isolated from raw milk. Briefly, raw milk samples were acidified, filtered, and centrifuged to remove debris. The resulting supernatant was treated with chloroform to eliminate bacterial contamination. The supernatant was mixed with K12, the host bacterium, and the presence of bacteriophages was verified using the double-layer agar method. Single plaques were isolated to ensure the purity of the bacteriophages. Optimal multiplicity of infection (MOI) was determined to facilitate efficient bacteriophage propagation. The characterization of the bacteriophages was conducted through a series of experiments. Host range determination was employed by using spot tests against various bacterial strains, and the bacteriophage genome was analyzed via restriction fragment length polymorphism (RFLP) to identify unique restriction profiles. Replication parameters, including the latent period and burst size, were derived from one-step growth curves, and adsorption rates were calculated to understand bacteriophage-host interaction efficiency. Morphological characterization was done using transmission electron microscopy (TEM) to identify the bacteriophage based on its tail and head structure. Additionally, the stability of the bacteriophage was evaluated under various pH and temperature conditions to assess its robustness for potential applications.^17^

For the preparation of new bacterial stocks, 50 μL of bacterial culture was inoculated into 5 mL of TSB and incubated overnight at 37°C. After incubation, 100 μL of the liquid culture was spread onto EMB agar and incubated at 37°C for 18–24 h to obtain discrete colonies. A single colony was picked using a sterile loop and transferred into 5 mL of fresh TSB. The resulting culture was incubated under the same conditions to produce a uniform bacterial suspension. 50 μL of K12 was inoculated into 5 mL of TSB and incubated at 37 °C for 18–24 h. After incubation, 1 mL of the culture was transferred to an Eppendorf tube and centrifuged at 4,500 × *g* for 5 min. The supernatant was discarded, and the pellet was resuspended in PBS. The bacterial counts were determined using EMB agar by spreading appropriate dilutions of bacteria onto the agar. After incubation at 37 °C for 18–24 h, colonies were counted. For propagation of the bacteriophage, the log-phase bacterial culture (OD_600_: 0.5–0.6) was mixed with K12.4a bacteriophage at a MOI of 0.01. The mixture was incubated for 6 h to allow bacteriophage replication. Then, the mixture was centrifuged at 12500 × *g* for 6 min at 4 °C, and the supernatant was filtered using a 0.22 μm syringe filter. The bacteriophage titer was determined using the double-layer agar method.^18^

### Synthesis of S1 and S2

In a standard preparation, 20 mg of **S0** was dispersed in 8 mL of Britton–Robinson pH 7 buffer solution and 2 mL of anhydrous acetonitrile. Subsequently, sulforhodamine B was introduced into the suspension and agitated at ambient temperature for 24 h to load the pores of the microparticles (**S1**). Following this loading step, a volume of 300 μL of bacteriophages K12.4a with a concentration of 10^9^ PFU/mL was added to **S1,** and the mixture was stirred for an additional 6 h. After 6 h of mixing, the particles (**S2**) were washed three times with distilled water at 5000 *g* for 10 min, and the remaining solid **S2** in the centrifuge tube was dispersed in 3 mL of distilled water.

### Dye Release Studies

A 1.5 mL portion of the **S2** solution was added to glass vials. The blank solution vial was completed with 3.5 mL of water, while the sample vials were completed with 500 μL of bacterial samples and 3 mL of water. Throughout the experiment, 150 μL samples were taken from the continuously mixed solutions at various time points. These samples were centrifuged at 10000 *g*, and the fluorescence emissions of the supernatants were measured at different time intervals at 586 nm after excitation at 565 nm.

### Point-of-Care Testing Device

The PoCT device was designed by the integration of S2 into a simple injector application. A reusable cartridge was fabricated and attached to the tip of the injector. The cartridge is composed of two cylindrical barrels accommodating the glass fiber filter between them (Figure 2). Both the top and bottom barrels were manufactured by directly machining cylindrical polyimide stock. The internal features, comprising a stepped hole, were machined by drilling and boring successively using a manually operated universal lathe (OPTIMUM D320 × 920, Optimum Maschinen GmbH, Hallstadt, Germany). To ensure axial alignment of the barrels during assembly, two alignment pins were snugly fitted into holes drilled on the face of the top barrel. During assembly, these alignment pins match with the alignment holes on the bottom barrel, ensuring the axial alignment of the pieces. The barrels, with the glass fiber filter in between, are secured by using a binder clip attached to the tabs machined on the top and bottom barrels. Tabs and alignment holes were machined using a manually operated universal milling machine (FOREMAN KF 360 A, Sezginler Makine, Bursa, Türkiye). To prevent leakage during operation, an O-ring was placed in the O-ring seat machined on the bottom barrel.

**Figure 2:**
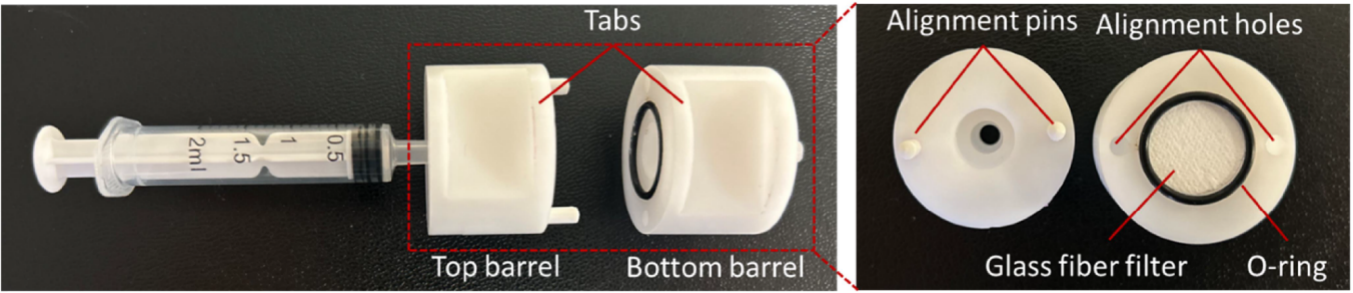
PoCT device components.

For the application of the samples, 1 mL of **S2** solution was drawn into a sterile injector with a total volume of 2 mL. Then, 500 μL of the sample was drawn into the injector. An equilibration period of 30 s was implemented to facilitate interaction between the bacteriophage-gated particle and bacteria. Subsequently, the solution was introduced into a filtering system, which featured a centrally positioned glass fiber filter. Fluorescence analysis of the resultant filtrate was performed at 586 nm using a microplate reader.

### Application to Lake Water Samples

Four different water samples were tested using a bacteriophage-gated sensor. Three samples were collected from small lakes in the Ostim region of Ankara, Türkiye. These lakes are polluted by industrial and domestic wastewater and are suspected of having a high load of bacteria. Another sample was collected from Sapanca Lake in Sakarya, Turkey. This water is used as a source of drinking water in the region. An ultrapure water sample was used as a blank sample, and all samples were tested with a routine bacteria test for comparison.

## Results & Discussions

### Synthesis and Characterization of the Sensory Material

Mesoporous silica nanoparticles with sizes around 100 nm were synthesized as described in the Experimental Section (Figure S1, Tables S1 and S2). Then, after filling the pores with Sulforhodamine B dye, two different mechanisms of bacteriophage immobilization on the silica surface were tested: physisorption and covalent binding with a linker, APTES. Physisorption was simply carried out by mixing dye-filled silica nanoparticles with bacteriophage solutions for 6 h. For covalent immobilization, APTES was first grafted onto the silica nanoparticles^19^ (Tables S1 and S3) and then, using EDC/NHS crosslinkers, bacteriophages were immobilized on the surface of the silica nanoparticles. Characterization of the mesoporous silica nanoparticles and their modifications is shown in the Supporting Information file. However, the gated mesoporous silica nanomaterials obtained through both immobilization methods did not produce a significant response to target K12 bacteria samples. A potential issue with these designs is that the bacteriophages may not effectively function as gates for the nanoparticles, as their size is comparable to that of the silica nanoparticles (ca. 100 nm).

Then, the size of the silica particles was increased from the nanometer to micrometer scale, and their shape was modified from spherical nanoparticles to more irregular forms (Figures S2 and S3). Moreover, the zeta potential of the particles was adjusted to near neutrality (∼−1 mV) by carrying out partial calcination to facilitate the physisorption of bacteriophages, enabling the creation of a functional gate for the sensor. The bacteriophages are weakly adsorbed on the neutral silica surface via physisorption. Upon contact with , they displace to adsorb to the surface of the bacteria and inject genetic material, which results in their dissociation from the support material and initiates dye release. The bacteriophage detachment from the silica surface is attributed to the natural infection mechanism. This interaction triggers the release of sulforhodamine B dye, generating a strong fluorescence response (*vide infra*). The sensor design (final solid **S2**) is illustrated in Figure 1.

### Main Characteristics of Bacteriophage K12.4a

In a previous study, the bacteriophage K12.4a was isolated, and its key characteristics were identified.^17^ In brief, the bacteriophage underwent purification after isolation to confirm the purity of the resulting plaques. Following the purification steps, the host range of the bacteriophage was tested using a spot test, which demonstrated that the bacteriophage was specific to its host bacteria.

For a comprehensive understanding of bacteriophage-host interactions, the replication parameters of the bacteriophage were established through adsorption and one-step growth curves. The burst size, latent period, and adsorption rate constant were calculated as 24 PFU/cell, 10 min, and 1.78 ± 0.60 × 10^– 8^ mL/min, respectively. Besides that, the stability of the bacteriophage to pH and temperature is a critical factor, as it plays a vital role in the effectiveness of all applications involving bacteriophages. It was observed that the titers remained stable within a pH range of 4 to 10 and a temperature range of 30 to 60°C.

Using the fragment profiles obtained from RFLP assays, the genome size of K12.4a was predicted to be 78.1 kb. Finally, the bacteriophage was imaged morphologically using TEM electron micrographs, revealing it to be a myovirus with the following dimensions: 17.27 ± 2.12 nm × 93.20 ± 9.21 nm (tail) and 92.84 ± 0.58 nm × 68.76 ± 2.03 nm (head) (Figure S4).

### Characterization of the Bacteriophage-Gated Optical Sensor

N_2_ adsorption-desorption, SEM, EDX, transmission electron microscopy (TEM), FTIR, XRD, and TGA were used for the characterization of the sensing material (**S2**).

The XRD patterns of silica microparticles show four diffraction peaks, which can be indexed as the Bragg peaks (100), (110), (200), and (210) and are characteristic of a hexagonal array of pores. As expected, the peak at (100) shifted during the calcination process because of the condensation of the silanol groups. The intensity of the (100) peak decreased, while the remaining peaks broadened, indicating a loss of contrast caused by filling the solid with dye and capping the pores with bacteriophages (Figure 3A).

**Figure 3:**
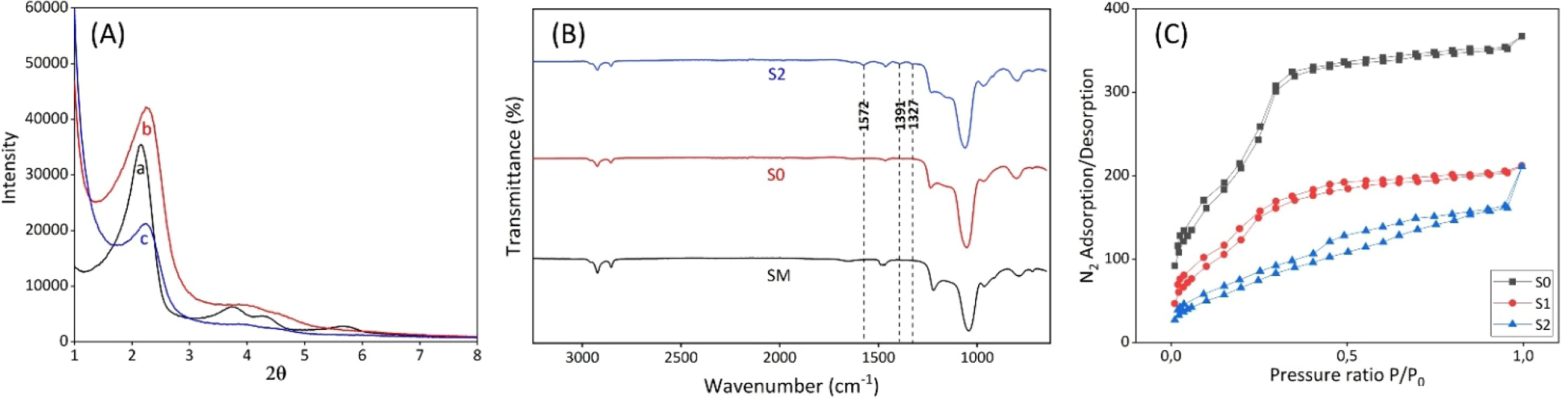
(A) XRD spectra of a) **SM** (silica microparticles as made), b) **S0** (partially calcined silica microparticles), c) **S2** (phage-gated silica microparticles). (B) FTIR spectra of **S0**, **S1**, and **S2** (phage-gated microparticles). (C) N2 adsorption/desorption curves for **S0**, **S1**, and **S2**.

In the FTIR study, signals of particles before calcination (**SM**), after partial calcination (**S0**), and bacteriophage-capped (**S2**) were analyzed (Figure 3B). Characteristic bands originating from Si–O–Si groups were observed in all three particle samples.^20^ As anticipated, spectral differences were evident between the bacteriophage-functionalized particles and the other particles. The peaks observed in the range of 1300–1600 cm^–1^ were attributed to bacteriophage origin and indicated DNA/RNA and protein structures derived from bacteriophages.^21^ The bands originating from bacteriophages are denoted in Figure 3B.

The thermogravimetric analysis of sample S2 reveals a 3.46% mass loss between 0–200°C, attributed to the evaporation of absorbed water. A more significant mass loss of 36% occurs between 200–700°C, corresponding to the decomposition of organic components, including bacteriophages, dyes, and surfactant residues. Finally, a 14.06% mass loss in the 700–1000°C range is associated with the degradation of silanol groups present in the silica microparticles.^20,22^

The N_2_ adsorption-desorption isotherms of **S0** exhibit two adsorption steps (Figure 3C). The first step occurs at intermediate relative pressures (*P*/*P*_0_ = 0.1–0.4) and is attributed to nitrogen condensation within the mesopores via capillary action. The pore volume, calculated by using the BJH model on the adsorption branch of the isotherm, is 0.72 cm^3^/g. Application of the BET model yielded a total specific surface area of 1290 m^2^/g. In addition to the adsorption step associated with the micelle-generated pores, a second feature is observed at high relative pressures (*P*/*P*_0_ > 0.9). Significant reductions in surface area (793 m^2^/g for **S1** and 417 m^2^/g for **S2**) and adsorbed N_2_ volume (0.43 cm^3^/g for **S1** and 0.38 cm^3^/g for **S2**) correspond to the filling of pores with dyes and the capping of bacteriophages on the particle surfaces.

In EDX analysis, a high amount of carbon (62.35% in atomic percentage) in the bacteriophage-gated sensor (**S2**) compared to **S0** shows the immobilization of the bacteriophages on the silica surface (Figures S5 and S6).

TEM and SEM images (Figures S2 and S3) reveal that the obtained particles are within the targeted microscale dimensions, with sizes of 1.69 ± 0.31 μm. The N2 adsorption-desorption curve and the TEM image (Figures 3C and S2) show that the calcination process generates pores.

### Method Optimization and Application

A pH optimization study was conducted between pH 2–9 using Britton–Robinson buffers. The results show that pH 7 is optimal for the gated sensor (Figure 4a). This is probably due to the effective attachment-detachment process of bacteriophages on the almost neutral silica surface (ζ: −1 mV) and the high activity of bacteriophages targeting bacteria at this pH level.^17^

**Figure 4:**
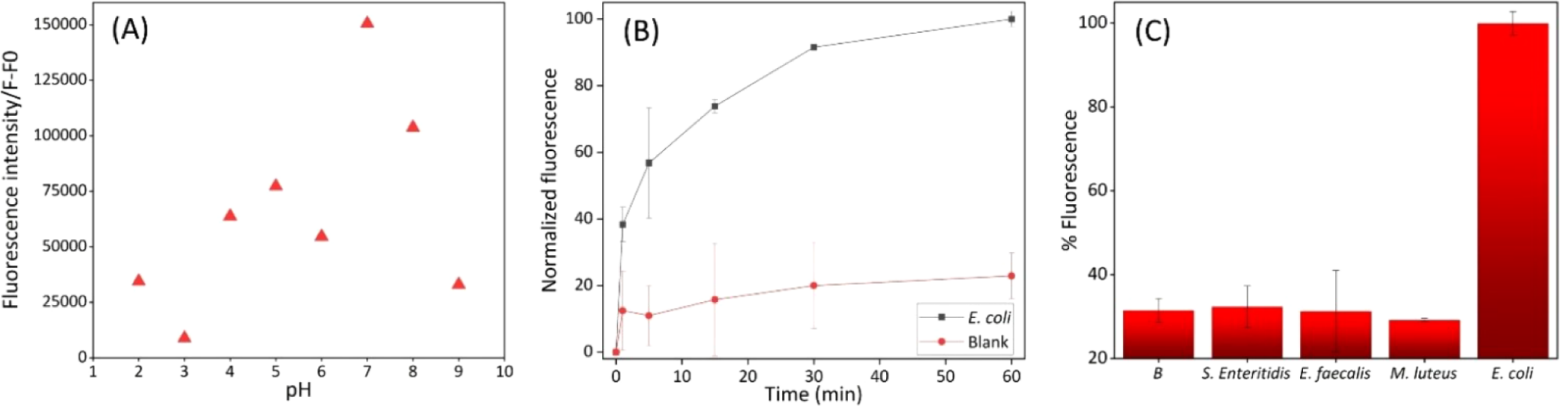
(A) Dye releases at different pH values in the presence of samples. (B) Release profile of sulforhodamine B from bacteriophage-gated sensor in the presence and the absence of , (c) Selectivity results: fluorescence response against different bacterial species.

Delivery kinetics were followed for 60 min by agitating **S2** and sample mixtures at room temperature and measuring fluorescence of the aliquots at different time intervals. The dye starts releasing just after the addition of , and the difference in emission signal between positive (presence of ) and negative samples increased over time (Figure 4b).

Selectivity of the sensor was tested against three different bacteria: , , and . Figure 4c shows that these bacteria were not able to deliver the cargo, whereas a selective release of the entrapped dye is observed in the presence of .

 samples at concentrations ranging from 10^1^ to 10^8^ CFU/mL were tested in vials, as described in the Experimental Section. The blank-subtracted results demonstrated that **S2** exhibited very high sensitivity, capable of detecting even 10^1^ CFU/mL, which shows a high blank-subtracted fluorescence signal (Figure S7). However, the quantitative performance of **S2** was limited, providing mostly qualitative YES/NO-type responses. Nonetheless, in most bacterial testing scenarios, detecting the presence of specific bacterial species is sufficient, with quantitative information rarely being required.

A PoCT device was developed following the ASSURED criteria, which emphasize affordability, sensitivity, specificity, user-friendliness, rapid and reliable performance, equipment-free operation, and deliverability to underserved populations.^23^ The device consists of a 2 mL sterile injector and a reusable cartridge, designed to accommodate interchangeable filters for each application (Figure 2). The fluorescence signal can be observed with the naked eye under a 405 nm LED light (Figure S8) or measured using a fluorescence spectrophotometer. Samples with concentrations ranging from 10^1^ to 10^8^ CFU/mL were also tested using the PoCT device, with results presented in Figure 5. The detection limit for the PoCT device was determined to be 10^1^ CFU/mL.

**Figure 5:**
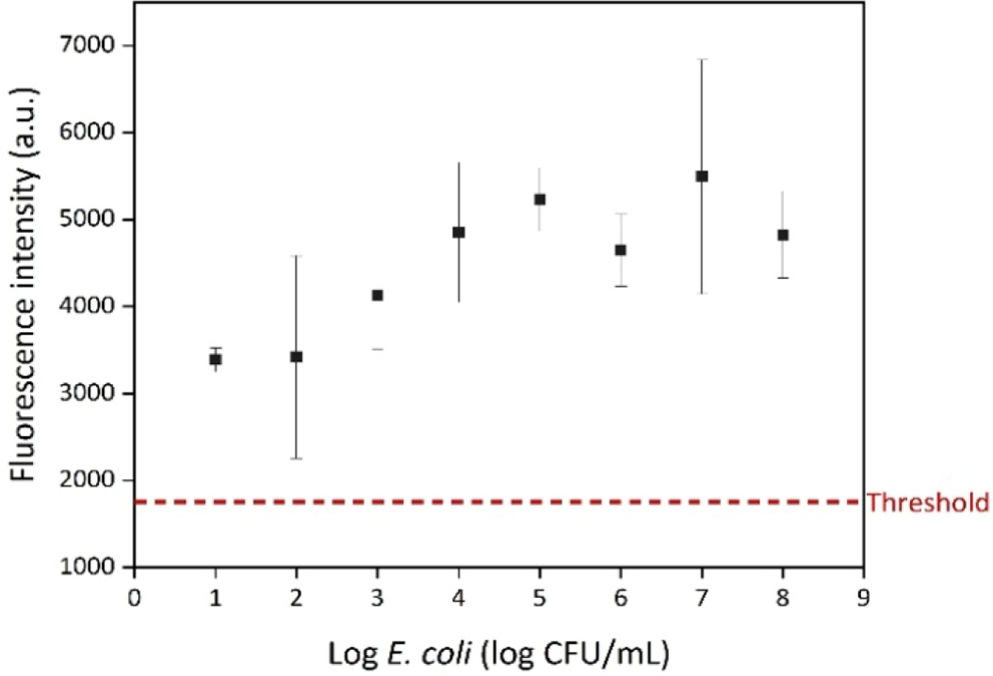
Results of PoC testing using the injector and cartridge on different concentrations of samples. The threshold was calculated from fluorescence intensities of ten blank solutions using the ″μ + 3 × SD” formula.

Four water samples (Sapanca Lake (WS1) and various wastewater sources (WS2, WS3, and WS4)) were tested for real sample applications, with three (WS2, WS3, and WS4) exceeding the detection threshold and one (WS1) falling below it (Table 1). The results were compared to routine bacterial testing. For this analysis, 100 μL of each coarsely filtered sample was inoculated onto sterile eosin-methylene blue agar using the surface spreading technique and incubated at 37°C for 24 h. After incubation, dark purple-black colonies with a metallic sheen, characteristic of , were counted to estimate the bacterial population (Table 1). All analyses were performed in triplicate. The results showed detectable loads in three samples, while the WS1 sample was below the threshold. The PoCT results were consistent with the culture-based method, confirming the accuracy of the bacteriophage-gated sensor in real-world applications.

**Table 1:** Real Water Sample Testing Results

	results	
Sample	Reference method (log CFU/mL)	Bacteriophage-gated sensor
WS1	BDL^a^	Negative
WS2	3.85 ± 0.21	Positive
WS3	2.77 ± 0.10	Positive
WS4	4.00 ± 0.00	Positive

a BDL: Below detection limit (<10 CFU/mL).

Table S4 summarizes the key performance parameters of the bacteriophage-gated sensor developed in this study, compared with various commercial detection kits. The sensor demonstrated a detection limit significantly lower than that of commercial tests that do not include enrichment steps. Although some commercial products are designed for use with different matrices, such as stool, food, and beverages, and achieve higher sensitivities through additional enrichment processes, these enrichment steps typically require approximately 1 day to complete. In contrast, the bacteriophage-gated sensor achieves a much faster processing time of just 5 min, compared to conventional methods that generally require 10 to 25 min. Furthermore, the estimated cost per test for the bacteriophage-gated sensor is considerably lower than that of the commercial kits. These results highlight the sensor’s high sensitivity, rapid response, and cost-effectiveness, making it a promising candidate for point-of-care applications, particularly in resource-limited settings.

## Conclusions

In conclusion, we report the design, synthesis, and characterization of a novel bacteriophage-gated sensor for the detection of . The sensor is loaded with the reporter dye sulforhodamine B, and its outer surface is functionalized with bacteriophages specific to the target bacteria. The sensing mechanism relies on the bacteriophages on the particle surface recognizing the target bacteria, detaching from the surface, and releasing the entrapped dye. This was validated through kinetic release experiments, which showed a significant increase in fluorescence response exclusively in the presence of . The PoCT device utilizing the bacteriophage-gated sensor meets the ASSURED criteria. This low-cost, reusable device delivers a highly sensitive and selective fluorescence response within 5 min. It is user-friendly and can be operated by nonprofessionals using a simple LED lamp. The accuracy of the bacteriophage-gated sensor was confirmed by testing real water samples and comparing the results with routine culture-based methods. To the best of our knowledge, this is the first study to employ bacteriophages as biological gates in a gated sensing platform. This approach differs from traditional gate-sealing methods, such as those using aptamers or antibodies, by offering enhanced environmental stability, biological specificity, and cost-effectiveness while maintaining responsiveness in complex matrices and enabling rapid, naked-eye detection. Moreover, the system can be adapted to incorporate other bacteriophages targeting different bacterial species. Future studies may explore multiplexing strategies by integrating diverse phage-capped particles into sensor arrays for simultaneous pathogen detection, as well as evaluating performance across various matrices relevant to bacterial testing.

## Supplementary Material



## Notes

XRD spectra, TEM images, SEM images, EDX results, N_2_ adsorption-desorption results, concentration study, photograph under LED light (PDF)
