# Supplementary material for: Bacteriophage-Gated Optical Sensor for Bacteria Detection
Source: Anal Chem. 2025 May 23;97(25):13103–9. doi: 10.1021/acs.analchem.5c00780 (PMC12224151; doi:10.1021/acs.analchem.5c00780)
Supplement: Supplementary file 1 [file ac5c00780_si_001.pdf]

SUPPLEMENTARY INFORMATION

Bacteriophage-gated optical sensor for *E. coli* detection

Busra Canan Aslan[a,b], Esra Ekiz[c], Emine Kubra Tayyarcan[c], İsmail Hakki Boyaci[c], Aysen Gumustas[b,d,e], Ender Yildirim[e,f], Ugur Tamer[e,g], Ebru Evren[h], Elcin Ezgi Ahi[i], Ramón Martínez-Máñez[j], and Mehmet Gokhan Caglayan[a]*

[a]*Ankara University, Faculty of Pharmacy, Department of Analytical Chemistry, Ankara, Türkiye*
[b] *Ankara University, Graduate School of Health Sciences, Ankara, Türkiye*
[c] *Hacettepe University, Department of Food Engineering, Ankara, Türkiye*
[d] *Ankara University, Faculty of Pharmacy, Department of Pharmaceutical Microbiology, Ankara, Türkiye*
[e] *ODTU MEMS Center, Ankara, Türkiye*
[f] *Middle East Technical University, Department of Mechanical Engineering, Ankara, Türkiye*
[g] *Gazi University, Faculty of Pharmacy, Department of Analytical Chemistry, Ankara, Türkiye*
[h] *Ankara University, School of Medicine, Department of Medical Microbiology, Ankara, Türkiye*
[i] *Gebze Technical University, Department of Chemistry, Kocaeli, Türkiye*
[j] *Instituto Interuniversitario de Investigación de Reconocimiento Molecular y Desarrollo Tecnológico (IDM), Universitat Politèc-nica de València, Universitat de València, Camino de Vera s/n, 46022 València, Spain*
*Email: gcaglayan@ankara.edu.tr

CONTENT PAGE

XRD Spectra and TEM images for mesoporous silica nanoparticles S2

Results of N_2 Adsorption_Desorption Analysis for silica nanoparticles S2

EDX results of silica nanoparticles S3

EDX results of APTES modified silica nanoparticles S3

TEM images of silica microparticles S3

SEM images of silica microparticles S4

TEM image of phage K12.4a S4

EDX results of mesoporous silica microparticles S5

EDX Analysis for phage-gated silica microparticles S6

Quantiation study in vials S7

Photograph under LED light S7

Performance comparison of the sensor with existing commercial *E.coli* detection tests S8

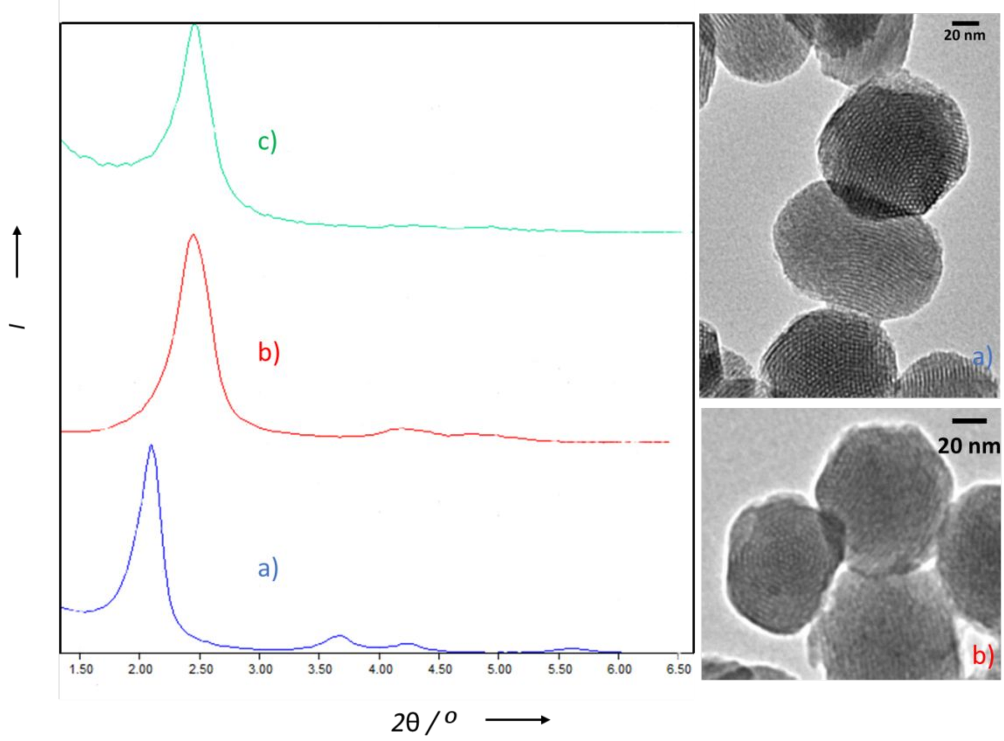

Figure S1. Left: Powder X-ray patterns of the solids a)MCM-41 as synthesized b) calcined MCM-41 silica nanoparticles and c) calcined MCM-41 silica nanoparticles containing the dye and functionalized with 3- aminopropyltriethoxysilane. Right: TEM images of calcined MCM-41 sample showing the typical hexagonal porosity of the MCM-41 mesoporous matrix and b) same particles containing the dye and functionalized with 3- aminopropyltriethoxysilane.

Table S1. Results of N_2 Adsorption_Desorption Analysis

Solid	Hydrodynamic particle diameter (nm)	Zeta potential (mV)	BET surface area(m^2/g)	BJH adsorption cumulative volume of pores (cm^3/g)
**MCM-41**	168.00 ± 63.12	-23.40 ± 5.03	1118.2893	1.009463
**APTES-MCM-41**	397.50 ± 78.10	11.20 ± 5.77		

Table S2. EDX Analysis results of MCM-41 silica nanoparticles

Element	Line type	Wt%	Wt% sigma	Atomic%
N	K series	0	0	0
O	K series	56.78	0.76	69.76
Si	K series	43.19	0.76	30.23
Cl	K series	0.02	0.13	0.01

Table S3. EDX Analysis results of APTES modified MCM-41 silica nanoparticles

Element	Line type	Wt%	Wt% Sigma	Atomic%
N	K series	2.03	0.30	2.87
O	K series	52.40	0.33	64.97
Si	K series	45.33	0.32	32.02
Cl	K series	0.24	0.04	0.13

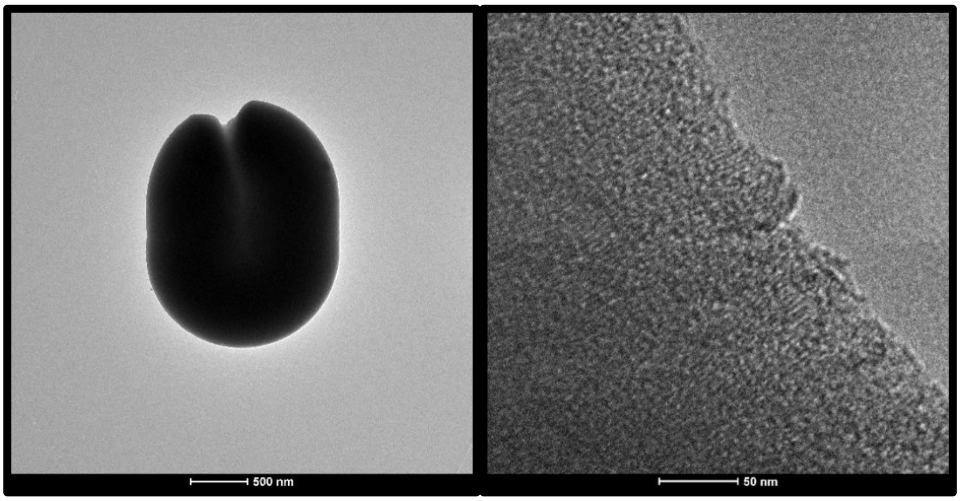

Figure S2. TEM images for silica microparticles showing size, shape and porosity of the particles

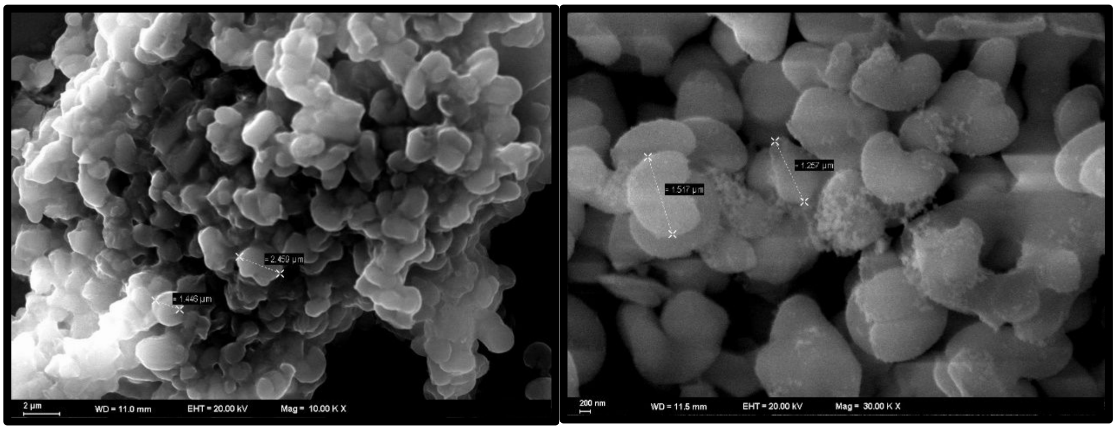

Figure S3. SEM images of silica microparticles and phage-gated silica microparticles (**S2**)

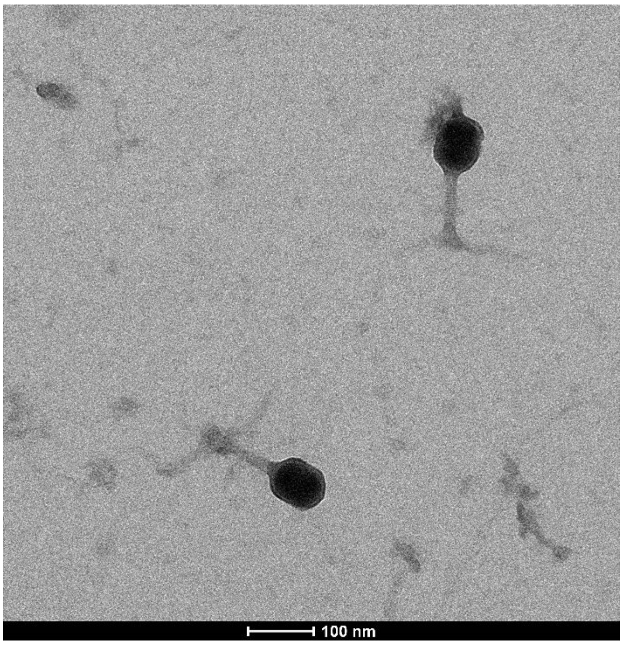

Figure S4. TEM image of phage K12.4a

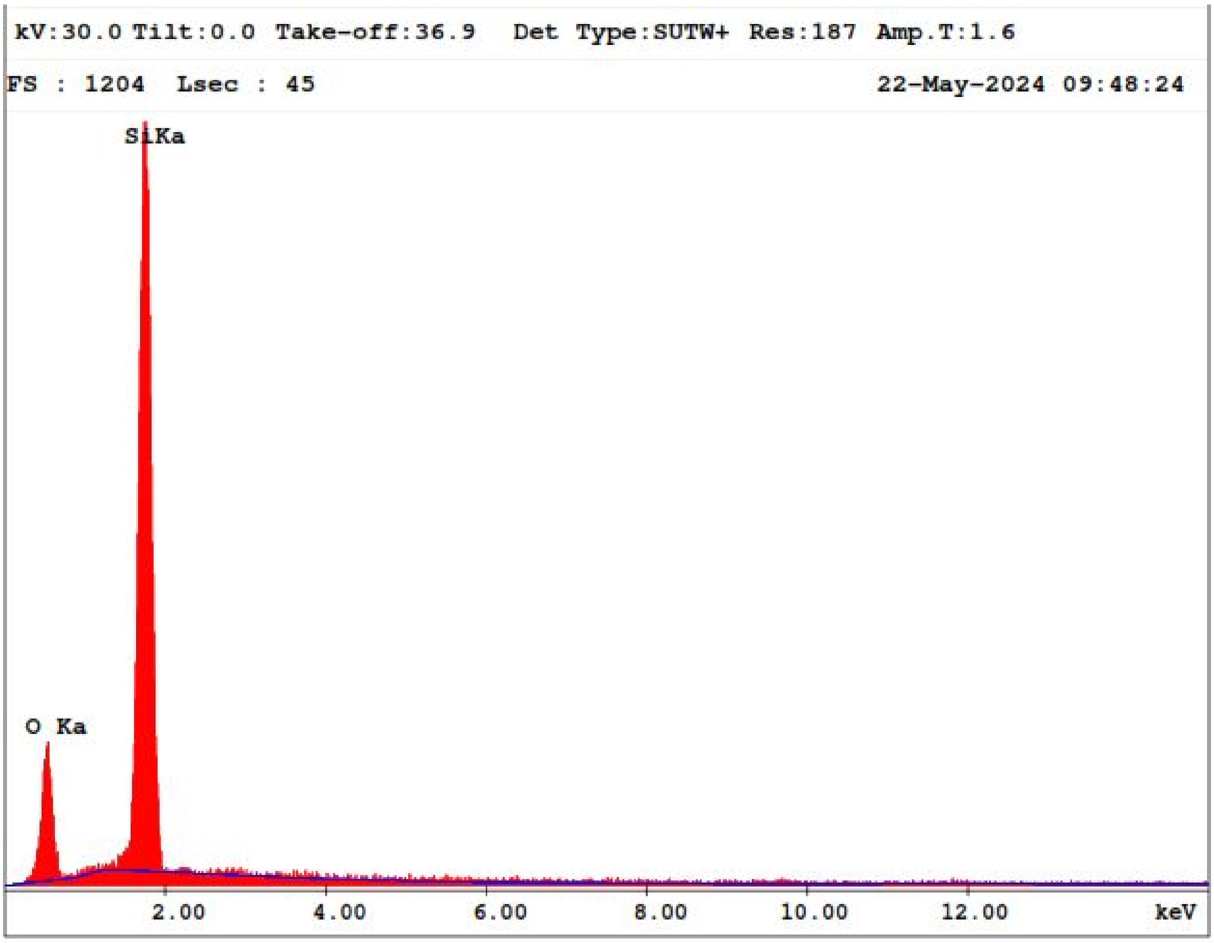

EDAX ZAF Quantification (Standardless)
Element Normalized
SEC Table : Default

Element	Wt %	At %	K-Ratio	Z	A	F
O K	46.48	60.39	0.1241	1.0189	0.2619	1.0007
SiK	53.52	39.61	0.3900	0.9832	0.7412	1.0000
Total	100.00	100.00				

Element	Net Inte.	Bkgd Inte.	Inte. Error	P/B
O K	56.96	2.24	2.05	25.45
SiK	360.03	8.20	0.80	43.90

Figure S5. Results of EDX analysis for **S0** (mesoporous silica microparticles)

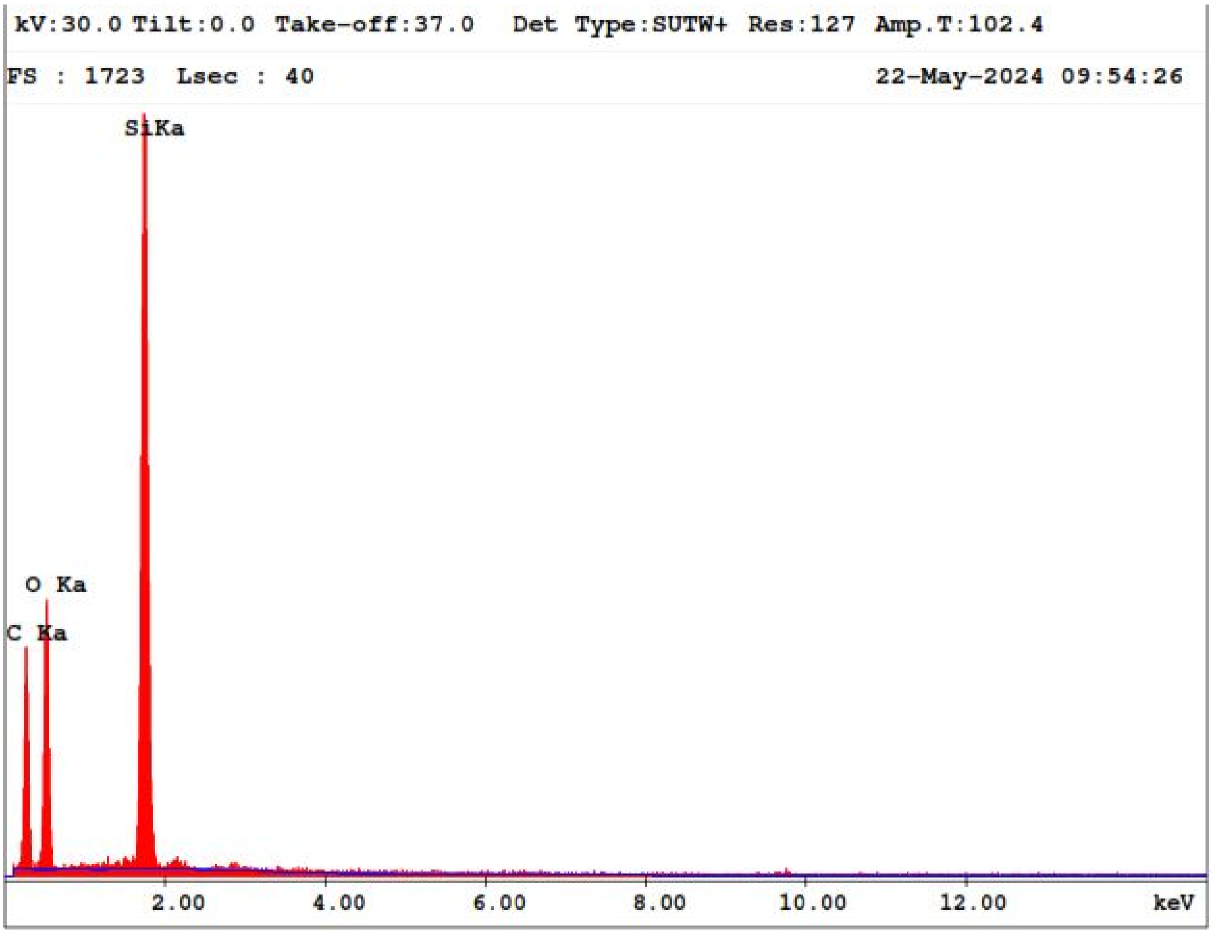

EDAX ZAF Quantification (Standardless)
Element Normalized
SEC Table : Default

Element	Wt %	At %	K-Ratio	Z	A	F
C K	51.89	62.35	0.1376	1.0117	0.2621	1.0003
O K	33.30	30.04	0.0550	0.9975	0.1654	1.0002
SiK	14.82	7.61	0.1088	0.9630	0.7623	1.0000
Total	100.00	100.00				

Element	Net Inte.	Bkgd Inte.	Inte. Error	P/B
C K	57.42	2.21	2.15	25.98
O K	75.74	2.06	1.85	36.71
SiK	301.46	3.32	0.91	90.93

Figure S6. Results of EDX analysis for **S2** (phage-gated silica microparticles)

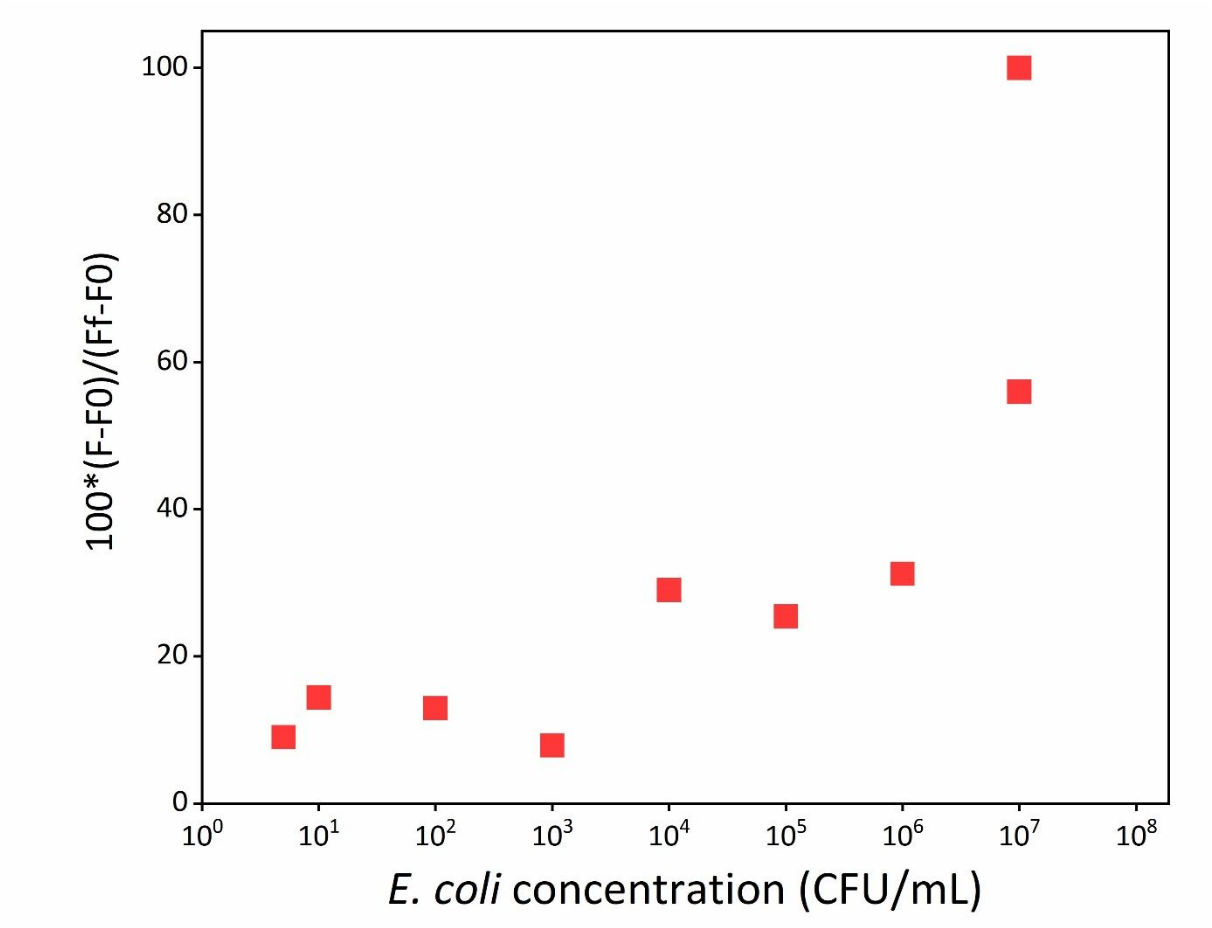

Figure S7. Blank subtracted and normalized fluorescence for samples with different *E. coli* concentrations.

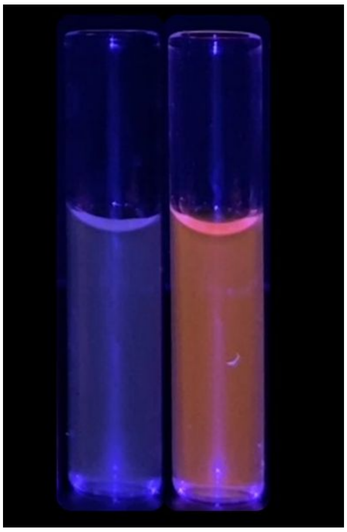

Figure S8. Photo of the blank (left) and E.coli (right) samples application under 405 nm LED light

Table S4. Performance comparison of the sensor with existing commercial *E.coli* detection tests

Product Name	Target E. coli Strain	Detection Limit	Processing Time	Estimated cost per test (€) *
Sigma Aldrich - Singlepath® E. coli O157	*Escherichia coli* O157:H7	1 CFU/25 g	24 hours for enrichment+ 20 minutes for test	~11
Meridian Bioscience - Immunocard STAT! ® E.coli O157 Plus	*Escherichia coli* O157:H7	8.3x10^5 CFU/mL	20 minutes	~43
Neogen - Reveal® 2.0 for E. coli O157:H7	*Escherichia coli* O157:H7	1 CFU/375 g	20 hours for enrichment + 15 minutes for test	~24
Romer Labs - RapidChek® E. coli O157 Test	*Escherichia coli* O157:H7	6 CFU/65 g	8–18 hours for enrichment + 10 minutes for test	~11
BioAssay Works - E. coli O157:H7 Rapid Detection Kit	*Escherichia coli* O157:H7	Not reported	~25 minutes	~12
BioControl Systems, Inc - VIP® Gold for EHEC	*Escherichia coli* O157:H7	Not reported	18–28 hours for enrichment + 10 minutes for test	~8
Certest Biotec S.L. - E. Coli O157:H7 detection kit	*Escherichia coli* O157:H7	1.87x10^4 CFU/mL	~10 minutes	~15
This study – Bacteriophage-Gated Sensor	*Escherichia coli* K12	10^1 CFU/mL	~5 minutes	~3

*The cost is estimated by the authors based on pricing in Türkiye and may vary in other countries.
